# Proportional Insulin Infusion in Closed-Loop Control of Blood Glucose

**DOI:** 10.1371/journal.pone.0169135

**Published:** 2017-01-06

**Authors:** Johan Grasman, Hannah L. Callender, Marco Mensink

**Affiliations:** 1 Biometris, Wageningen University and Research Centre, Wageningen, The Netherlands; 2 Department of Mathematics, University of Portland, Portland, Oregon, United States of America; 3 Division of Human Nutrition, Wageningen University and Research Centre, Wageningen, The Netherlands; Baylor College of Medicine, UNITED STATES

## Abstract

A differential equation model is formulated that describes the dynamics of glucose concentration in blood circulation. The model accounts for the intake of food, expenditure of calories and the control of glucose levels by insulin and glucagon. These and other hormones affect the blood glucose level in various ways. In this study only main effects are taken into consideration. Moreover, by making a quasi-steady state approximation the model is reduced to a single nonlinear differential equation of which parameters are fit to data from healthy subjects. Feedback provided by insulin plays a key role in the control of the blood glucose level. Reduced β-cell function and insulin resistance may hamper this process. With the present model it is shown how by closed-loop control these defects, in an organic way, can be compensated with continuous infusion of exogenous insulin.

## Introduction

This investigation deals with the modelling of a control mechanism by which, during and after a meal, the blood glucose level is maintained within safe bounds. For that purpose liver and skeletal muscle tissue are used as buffers. The method that is presented focuses on the control mechanism that allows such a configuration in which the hormone insulin plays a key role. The review article by Prentki et al. [1] gives a detailed account of the metabolic signalling that is involved in this process. However, based on such a description it is not possible to formulate a dynamical model for the concentration of blood glucose. Such a model has to represent the dynamics in a quantitative form and must be sufficiently robust. In this study we explore the use of a differential equation model to capture the dynamics of blood glucose by fitting it to data. This way of data-based modelling leads to a result that can be applied in the closed-loop control of the blood glucose level by an external input of insulin in case of diabetes [2–6]. The feedback algorithm that is proposed leads to coherent fluctuations of glucose and insulin in the blood circulation similar to those of nondiabetic persons.

The model we introduce consists of three differential equations. Input variables are the glucose component of a meal and the energy expenditure in cells from different body tissues. During digestion of a meal glucose passes through the wall of the gastrointestinal tract and enters the blood circulation. Due to the large intake of glucose during a meal it is expected that the inflow into the bloodstream has a temporal pattern similar to the blood glucose level itself. Therefore, the time dependency of the inflow is assumed to have a particular type of distribution resembling graphs from the literature [7]. The Gamma distribution fits such a profile very well. The values of the parameters that describe the inflow of glucose from a meal are found together with the other parameters when fitting the blood glucose profile to available data. The differential equation approach of blood glucose dynamics has been presented in a number of studies [8–10]. In [2] an overview is given that starts with the Bergman minimal model [11], which consists of a system of three differential equations with variables denoting glucose and insulin concentrations in different compartments.

Normally, the amount of glucose in the blood circulation is tightly kept within a fixed interval. For a healthy person with a regular eating pattern blood glucose levels vary between ~4 (fasting) and up to 10 mmol/L (postprandial). During a meal a strong glucose pulse enters the blood circulation. At its peak the flow can be at a rate of more than 250 mmol/h within a short time interval of less than an hour. The hormone insulin, produced in the β-cells of the pancreas, controls glucose homeostasis. It facilitates the uptake of glucose into tissues and stimulates the oxidation of glucose above fats. Moreover, it takes a role in the storage of glucose in liver and skeletal muscle tissue in the form of glycogen. The reverse process of raising blood glucose levels is mainly steered by a decrease in insulin and an increase in the counter regulatory hormone glucagon.

The process of glucose control in the blood is modelled by a dynamical system consisting of three variables [12]: *x* being the concentration of glucose in blood, *y* the concentration of insulin and *z* the concentration of glucagon. The interactions of these variables are assumed to be of the type of low order chemical reactions. Instead of describing the dynamics on the basis of what is known about the precise processes underlying the way in which the glucose flow within the body is steered, we concentrate on the direct interactions between the three system variables leading to a description of the system as we observe it. It is anticipated that this description covers the main processes in the control of the blood glucose level. Most importantly the model is required to give a sufficiently accurate quantitative description of the blood glucose profile during the intake of a carbohydrate rich meal. Various simplifying assumptions are made: liver and muscle tissue may store an unlimited amount of glucose, while through the liver an unlimited amount may be delivered. Furthermore, conversion of proteins into glucose and of protein and glucose into fats are secondary processes that are not taken in consideration; they do not play a decisive role in the handling of a large intake of glucose during and shortly after a meal [9].

Whence we have composed such a differential equation model, we start making an approximation by assuming that the system is in a quasi-steady state [13]. Such an approach can be justified if the dynamics of the variables have different time scales [14–15]. This results in a single differential equation for the blood glucose concentration with parameters that are fitted to available data. To further simplify the model some of these parameters are the result of lumping and have no direct physical meaning. Our goal is to develop a model that correctly describes the blood glucose profile during a meal and the return to the baseline after the meal. Other conditions such as heavy exercise by a subject [16] are not covered; hormones such as adrenalin would completely change the blood glucose dynamics and are not considered in this model. Insulin-independent glucose uptake by cells is also not taken in consideration. In this way we arrive at a minimal model for a single compartment with only the glucose concentration in the bloodstream as variable.

## Materials and Methods

### The model

Glucose, variable *x*, enters the blood circulation in two ways: from digested food containing carbohydrates and from the liver where it was stored as glycogen (‘glycogenolysis’). Glucose may also be newly synthesized from precursors like certain amino acids and glycerol (‘gluconeogenesis’). During an overnight fast low insulin levels favour hepatic glycogenolysis and hepatic and renal gluconeogenesis [17]. We neglect renal glucose production and consider only the glucose inflow from digesting a meal and glucose that is released from liver, i.e. hepatic glucose production which combines both glycogenolysis and gluconeogenesis. This last flow is assumed to be proportional with the concentration of glucagon in the blood, with proportionality constant ε. This condition may hold if the glycogen level in liver is sufficiently high. Glucose leaves the blood circulation to be used for energy expenditure in cells and by being stored in liver and muscle tissue (‘glycogen synthesis’) when the blood glucose level is high. This last process can be modelled as a second order chemical reaction leading to a decrease of blood glucose at a rate δ*xy*, in which the coefficient δ is related to insulin resistance *R* = 1/ δ [18]. The dynamics of glucose thus reads

$$\frac{dx}{dt}=I(t)-E(t,x)-\delta xy+\varepsilon z,$$ (1)

where *I*(*t*) denotes the inflow of glucose in the blood circulation through the wall of the gastrointestinal tract and *E*(*t*, *x*) the transformation of blood glucose into energy that is expended by the body.

Insulin, variable *y*, is produced by the β-cells in the pancreas at a rate that is assumed to depend linearly upon the blood glucose level. Insulin is cleared from the blood circulation by liver and kidneys at a constant rate. Furthermore, insulin decays linearly in blood with a half-life in the order of 5 minutes. Matthews et al. [18] modelled the homeostatic relation between blood glucose and insulin concentrations at low glucose concentrations with the insulin concentration *y* in mU/L. Using their result we formulate a differential equation for the dynamics of insulin:

$$\frac{dy}{dt}=\beta b(x,x_{0})-\lambda y\;with\;b(x,x_{0})=\max(0,x-x_{0}),$$ (2ab)

where the decay parameter takes the value λ = 8.3 h^-1^. The parameter β represents the β-cell function. In [18] a value of 3.5 mmol/L in plasma is found for *x*_0_. Taking into account that our data is in whole blood we set *x*_0_ = 3 mmol/L. Furthermore, from Fig 1 of [18] it is found that at a 100% β-cell function β/λ = 4.2 giving β = 34.9 mU h^-1^mmol^-1^.

Glucagon, variable *z*, is produced by the α-cells in the pancreas. A low blood glucose level corresponds with an increased glucagon production. In [19] it is stated that insulin plays an intermediating role in this respect: a high insulin production inhibits glucagon production by α-cells. For the expression of the production of glucagon as it depends on the insulin production we take an exponential function. It requires only one parameter (*m*) and exhibits a close to linear decay. Moreover, for larger values of the insulin production it will tend to zero while remaining positive. This leads to a differential equation for glucagon dynamics:

$$\frac{dz}{dt}=\alpha d(\beta b(x,x_{0});m)-\gamma z\;with\;d(\beta b(x,x_{0});m)=\exp(-\beta b(x,x_{0})/m).$$ (3ab)

The parameter α denotes the maximal production of glucagon by the α-cells. Glucagon has similar to insulin a half-life on the order of minutes; the parameter γ represents this process of decay. The expression (3b) indicates that glucagon production takes larger values when the insulin production is decreasing at lower glucose concentrations.

For the function *I*(*t*) the profile of a Gamma-distribution is taken with a multiplicative factor *w* denoting the total amount of glucose in the meal:

I(t;w,a,k)=wGamma(t;a,k). (4)

It is assumed that during digestion the expenditure of calories is constant. Moreover, the fraction of expended glucose based calories depends on the blood glucose concentration: the higher the concentration the larger the fraction. The following functional relation is taken

$$E(x;p,q)=q\{1-\exp(-p(x-x_{0}))\}\;with\;p=0.7,$$ (5)

so that at the upper boundary of feasible blood glucose concentrations, *x* = 10 mmol/L, the fraction of glucose based expended calories is about 99%. The dynamics of (1) is steered by the forcing term *I*(*t*; *w*, *a*, *k*) so it acts at a time scale of hours. The dynamics of (2) and (3) act at a time scale of minutes with half-lives on the order of 5 minutes [20–21]. This large difference in times scales between (1) and at the other hand (2) and (3) allows a quasi-steady state approximation [13] meaning that for (2) and (3) the equilibrium can be taken as an estimate for the solution, so

$$\beta b(x,x_{0})-\lambda y=0,\;\alpha d(\beta b(x,x_{0});m)-\gamma z=0.$$ (6ab)

### Fitting parameters from data

The variables *y* and *z* can be removed from (1) by using (6ab); this changes (1) into

$$\frac{dx}{dt}=I(t;w,a,k)-E(x;p,q)-\omega b(x,x_{0})x+\sigma d(b(x,x_{0});n)$$ (7)

with

ω=δβ/λ,n=m/βandσ=αε/γ. (8abc)

It is noted from (8a) that from blood glucose data the product of the parameters δ and β can be estimated using the estimate of ω. However, the individual parameters are not identifiable. If we leave out the intake of food and the effect of physical exertion, the dynamics of blood glucose is governed by the last two terms of the right hand side of (7). These make the system tend to an equilibrium *x* = *x*_*b*_, called the baseline. It is remarked that other hormones such as cortisol and adrenalin still may play a role and affect the estimation of the parameters, in particular σ, so that in models with more detail the values of these parameters will change.

Next we construct with (7) a blood glucose profile brought about by meal 1 given in Table 1 and Fig 5 of [7], where during digestion of a fast absorption meal venous blood glucose values were measured from 23 nondiabetic subjects. The meal had a total glucose content of *w* = 50.5 g, or 280.3 mmol. In the figure average values are given.

**Fig 1:**
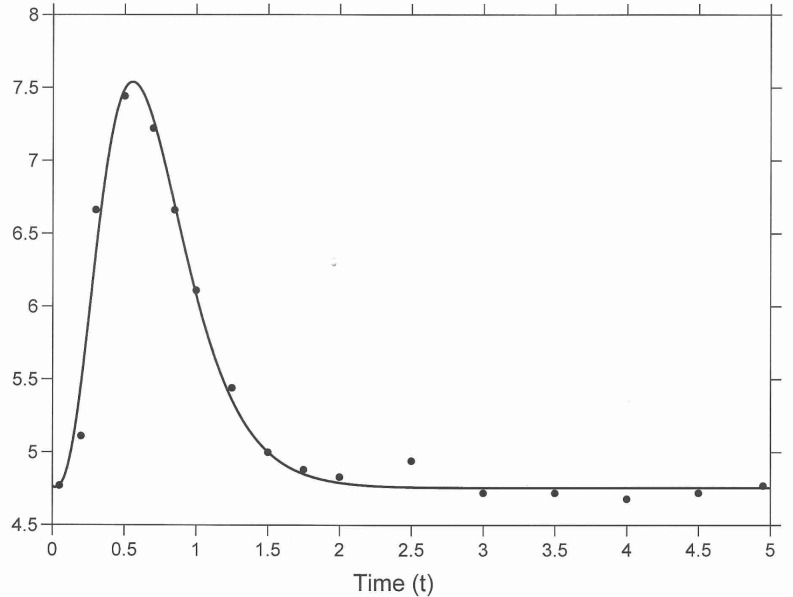
Fitting the solution of differential Eq (7). The data (o) is from a meal which has rice as the main component (Fig 5 of [7], ∆ Meal 1) with *w* = 280.3 mmol. The parameter estimates are given in (9).

We assume that *q* = 100 mmol/h being equivalent with a glucose based energy expenditure of about 75 kcal/h. For the parameter *n* we choose the value 1.75, see [22]. At the starting and end point of the measurements this system is in the equilibrium state meaning that the effect of the forcing *I*(*t*; *w*, *a*, *k*) is not felt. It corresponds with a baseline value *x*_*b*_ = 4.8 mmol/L. For given parameter values the solution of (7) is found by numerical integration. The parameters *a*, *k*, ω and σ are varied in order to find the values of these parameters for which the least square method yields the best solution. Using the Matlab based software package Grind (www.sparcs-center.org/grind) the best fit is found for

a=5.69,k=4.11,ω=8.30andσ=382, (9)

see Fig 1. Using (8a) it is concluded that at a 100% β-cell function (β = 34.9) the parameter δ = 2. We introduce the notion of insulin resistance by *R* = 1/δ = 0.5 h mU / L.

In S1 Appendix, a sensitivity analysis for the peak value and the base line has been carried out. Two methods were utilized: Pearson Rank Correlation Coefficients (PRCC values) and extended Fourier Amplitude Sensitivity Testing (eFAST).

## Results

### Dependence upon β-cell function and glucose content of the meal

**Fig 2:**
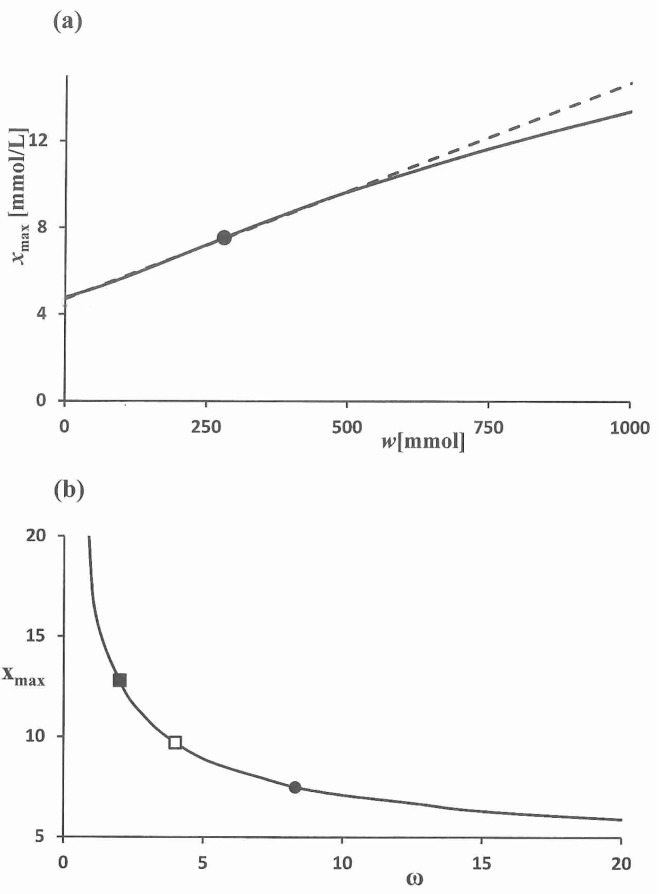
Peak value *x*_max_ as a function of the size *w* of the carbohydrate component of the meal and the parameter ω. (a) Parameter *w* is varied while the other parameters are fixed (solid line). The peak value that corresponds with the meal (*w* = 280.3) is given by (●). A linear regression (dashed line) is made after deleting the values *w* ≥ 500: *x*_max_ = 4.7 + 0.01 *w*. (b) Dependence of *x*_max_ upon ω. The value of *x*_max_ for (9) is given by (●). For ω = 2 the peak value is 12.8 (■); this can be brought down to 9.7 by doubling ω (□), see the section on closed-loop blood glucose control.

The dependence of the peak value *x*_max_ upon the size *w* of the carbohydrate component of the meal is worth to be consided over a larger range of these parameters. In Fig 2A the results are given. There is a linear relation that holds for *w* within the interval [0, 500]. For a meal with given value *w*^(*m*)^ this line is determined by two points: (*w*^(*m*)^, $x_{\max}^{\left(m\right)}$) and (0, *x*_*b*_), where *x*_*b*_ is the baseline value. Such a relation only holds for meals with a comparable composition of carbohydrates.

The dependence of *x*_max_ upon the parameter ω plays a main role in the control of the blood glucose level, see the sensitivity analysis in S1 Appendix. It is noted, see Fig 1, that a value of ω = 8.30 yields for the given meal a blood glucose concentration that stays within the safe interval [3, 10]; in Fig 2B the corresponding peak value is indicated by (●). In case the β-cell function falls short and a dosage of exogenous insulin is mandatory, it is important to know the decrease of the peak *x*_max_ as a function of the amount of exogenous insulin. In case of diabetes (ω = 2) a peak value of 12.8 will occur for the meal that was presented (■). This can be brought down to 9.7 by doubling ω (□). In the next section it is worked out how this technically can be achieved.

### Closed-loop blood glucose control

In the case of diabetes exogenous insulin can be brought into the blood circulation in order to maintain a safe blood glucose level. The external control variable *u*(*t*) introduced in (2a) changes (6a) into

$$\beta b(x,x_{0})-\lambda y+u(t)=0,$$ (10)

so that (7) becomes

$$\frac{dx}{dt}=M(x,t)-\{\omega b(x,x_{0})+\kappa u(t)\}x+\sigma d(b(x,x_{0});n)$$ (11)

with

M(x,t)=I(t)−E(x,t),u(t)≥0andκ=δ/λ. (12abc)

There is a vast literature on the control problem of finding the function *u*(*t*) that keeps the blood glucose concentration at a safe level [2, 23–24]. One group of investigations MPC (Model Predictive Control) takes a mathematical model as a starting point [4–6]. The nonlinear model (11) could be used for that purpose in a control algorithm such as the Kalman filter [25]. However, being aware that a high blood glucose level is due to a low value of the parameter ω, a natural choice is to take

$$u(t)=\rho b(x(t),x_{0})$$ (13)

with ρ ≥ 0 and *b*(*x*, *x*_0_) given by (2b). It is noted that from the total inflow of insulin in the blood circulation at all times a fixed fraction

μ=ρ/(ρ+β) (14)

comes from the infusion device. This type of external feedback is similar to the power control mechanism of the pedelec (electric bicycle). The approach differs from control algorithms such as PID (Proportional-Integral-Derivative control) that use a set point, e.g. the baseline [5–6]. If our model were perfect, then ρ could be kept at a constant value being sufficiently large to let the blood glucose concentration stay at all times within the safe range. Moreover, the body will experience coherent fluctuations of glucose and insulin in the blood circulation comparable with those of a nondiabetic person. This may bring about favourable effects such as a reduced risk of (further) insulin resistance [26]. If the infusion consists of long-acting insulin with a slower decay in the blood circulation (λ_1_ < λ), then (13) changes into

$$u_{1}(t)=(\lambda_{1}/\lambda )\rho b(x(t),x_{0}).$$ (15)

Additional functions can be incorporated in the insulin infusion device, such as range control by cutting off dosage below a certain blood glucose level and a temporal increase of ρ at extremely high blood glucose values. For β > 0 fine tuning can also be done by letting the amplification factor μ depend upon *x* and its time derivative $\dot{x}$:

$$\rho (x,\dot{x})=\frac{\mu (x,\dot{x})}{1-\mu (x,\dot{x})}\beta .$$ (16)

By increasing the amplification factor for $\dot{x}$ > 0 the system will anticipate a larger need of insulin and compensate for delays in the control system.

In order to further work out the closed-loop control for a diabetic case, we take the case (9) with now ω = 2, assuming that there is no insulin resistance. Thus, we have δ = 2, so that κ = 0.24 and β = 8.3. The blood glucose peak value of 12.8 in Fig 2B is brought back to 9.7 (ω = 4) for ρ = 8.33. Thus, in that case the insulin infusion has to be at about 25% of the production by the pancreas of a healthy person. For meals with a different value of *w* the value of ρ, that brings the peak value *x*_max_ below 10 mmol/L, can be found by using the linear dependence of the peak value upon the size *w* of the meal (Fig 2A).

The parameter κ contains the unidentifiable parameter δ. This problem is solved by repeating the intake of a meal, e.g. the one with parameter values (9), with an external control (13) for which ρ takes a given fixed small positive value. Then the new estimate of the parameter ω will take a larger value ω_*c*_, so κ = (ω_*c*_*—*ω)/ρ. This yields with (12c) a value for the insulin resistance *R* = 1/δ.

## Discussion

The model we presented applies to blood glucose profiles that cover the intake of a carbohydrate rich meal. The profile starts at the baseline and returns to this value after the meal has been digested. The restricted amount of glucose that is allowed in the blood steers a flow of glucose to liver and skeletal muscle tissue. This flow depends in a nonlinear manner upon the amount of glucose in the blood circulation: the quadratic term in (7) with coefficient ω yields the required feedback to properly keep the blood glucose level below a critical high value. When the blood glucose concentration tends to get low, the flow is reversed. In this study the entire process is described by a single differential equation. This equation is derived from a set of three coupled differential equations for the concentrations of glucose, insulin and glucagon in the blood circulation. Simplifying assumptions have been made: the reactions of these three types of molecules are of low order and the fast reactions, at a time scale of minutes, are close to their equilibrium (quasi-steady state approximation). It is assumed that during a constant calorie expenditure at high glucose concentrations a higher fraction of glucose related calories will be expended compared with the expenditure at a low concentration level. Furthermore, only blood glucose profiles with one peak are taken into consideration. In the case of meals consisting of different types of carbohydrates, e.g. fructose or starch, glucose may enter the circulation with a more widely spread temporal distribution which may even have more than one peak [27].

The coefficients of (7) were fit to a blood glucose profile of nondiabetic subjects [7] yielding satisfying accuracy, see Fig 1. The data consists of glucose concentrations that have been averaged over all subjects. If individual time series were available, then the distribution of the subjects over the parameter space could be obtained. The resulting model can be used to predict the size of a glucose peak during a meal with a given amount of carbohydrates. This prediction takes the form of a linear algebraic equation (see Fig 2A). The accuracy of this prediction is limited because the distribution of glucose inflow may differ from meal to meal.

The parameter sensitivity analysis of the peak value (S1 Appendix) shows that indeed the term with ω in (7), that represents the feedback provided by insulin, plays a key role. A similar parameter sensitivity analysis of the baseline (S1 Appendix) shows that the term with σ, representing the production of glucagon, contains parameters that have a strong influence. Since the model does not allow other mechanisms by which glucose is brought back into the circulation, this term also covers other processes having the same effect. Because of this and the high sensitivity to the coefficients in this term, a refinement of the model would be a useful next step when an accurate control of the baseline is required.

Furthermore, it is noted that the differential Eq (2ab) for insulin is based on the assumption that the glucose concentration is low [18]. This means that the linear expression (6a) needs a correction for higher concentrations. For that purpose an additional parameter should be introduced. However, such an extension is not expected to yield a better fit, taking into account the increase in the number of parameters. A more useful next step would be to base the model on data containing concentrations of glucose as well as insulin. Then the quasi-steady state assumption for the insulin dynamics should be dropped. In that case separate estimates of β-cell function (β) and insulin resistance (*R*) can be made. A first exploration of such a follow-up project has been made. It shows that a sufficiently large number of data points are needed given the number of parameters that have to be estimated.

The study of closed-loop blood glucose control requires expertise of two almost distinct fields of science: the physiology of varying glucose levels and the construction of control algorithms. The latter is mostly based on a minimization of some cost function using mathematical techniques [2, 6]. In our study we base the algorithm on the feedback mechanism that is already present in the model of the physiological process. Our differential equation model is based on only three variables. It is therefore a highly simplified representation of the process of blood glucose control in the body as it is known up to now. This process is inherently nonlinear. Modelling this nonlinear component correctly is a necessary condition in order to accurately fit data of carbohydrate rich meals with a large glucose peak (data-based modelling). Having reached this goal, we obtained a robust system that also allows a robust closed-loop control [3, 28] in the form of proportional insulin infusion. Refinement of this control as suggested in the text needs to be supported by experiments in a clinical setting.

## Supporting Information

S1 Appendix (DOCX)
