# Supplementary material for: Proportional Insulin Infusion in Closed-Loop Control of Blood Glucose
Source: PLoS One. 2017 Jan 6;12(1):e0169135. doi: 10.1371/journal.pone.0169135 (PMC5217952; doi:10.1371/journal.pone.0169135)
Supplement: S1 Appendix — (DOCX) [file pone.0169135.s001.docx]

**S1 Appendix**

**Sensitivity analysis**

In order to find out the sensitivity of the peak *x*_max_ (at *t* = 0.583 h) to the different parameters, two methods were utilized: the Pearson Rank Correlation Coefficients (PRCC values) and extended Fourier Amplitude Sensitivity Testing (eFAST) (see [A1,A2]). PRCC values have been shown to be the most effective measures when nonlinear but monotonic relationship exist between model inputs and outputs [A3], while eFAST, a variance based method, is one of the most reliable measures for models where the inputs and outputs exhibit nonlinear and non-monotonic relationships [A4, A5]. For a complete sensitivity analysis, it is recommended to use both methods when dealing with nonlinear models [A2]. PRCC values along with information on their p-values (to determine whether or not the correlations are significantly different from zero) are given in Table I for the parameter sensitivity of the peak value. First-order (*S_i_*) and total-order (*S_Ti_*) sensitivity indices, being the outputs of eFAST, are also reported alongside information on their corresponding p-values in Table I. First-order indices report the fraction of variance in the model output that can be explained by the variance in a model input, whereas a total-order index for a specific input reports the variance in model output that remains when all variance caused by every other model input is removed. For each parameter, values are generated near the given value in (9) by drawing numbers from a uniform distribution over an interval with lower and upper value being 0.5 and 1.5 times the value given in (9). From the generated points the PRCC values and eFAST sensitivity indices are computed.

Table I. PRCC values, first- and total-order indices, and their p-values for measuring the sensitivity of *x*_max_ to model parameters. Parameters were allowed to vary within +/- 50% of nominal values, and Latin Hypercube sampling was performed to obtain the sample space. Values followed by * have p-value < 0.05. PRCC values were obtained using a sample size of N = 1000. eFAST sensitivity indices were obtained from *N* = *N_s_***k***N_r_* model runs, where *N_s_* = 1025 is the number of search curves, *k* = 9 + 1 is the number of model parameters plus a dummy parameter for calculating p-values, and *N_r_* = 5 is the resampling size, see [A2] for more details.

| parameter | *w* | *a* | *k* | *q* | ω | *x*_0_ | *p* | σ | *n* |
| --- | --- | --- | --- | --- | --- | --- | --- | --- | --- |
| PRCC | 0.600* | 0.584* | -0.121* | -0.225* | -0.585* | 0.631* | -0.070* | 0.147* | 0.293* |
| *S_i_* | 0.198* | 0.138* | 0.044* | 0.024 | 0.171* | 0.170 | 0.001* | 0.006 | 0.019* |
| *S_Ti_* | 0.241* | 0.338* | 0.233* | 0.027 | 0.202* | 0.172 | 0.002* | 0.010 | 0.023* |

It is remarked that the sensitivity analysis applies to the two groups of parameters, the ones that have a fixed value and the ones that were varied. A large PRCC and/or sensitivity index is found for the fixed parameters *w* and *x*_0_. Since their values are rather accurate, there is no reason to vary them. A large PRCC is also found for the varied parameter ω, but that is what we expect as it is the coefficient of the nonlinear term that controls the peak value.

An analogous sensitivity analysis is carried out for the baseline value *x_b_* (see Table II). For the fixed parameters *q, n* and *x*_0_ large values for PRCC and/or the sensitivity indices were found. The parameter *n* is not known accurately, because it is a coefficient in the last term of, Eq.(7) that represents different physiological processes. The same argument holds for the varied parameter σ being part of the same term.

Table II. PRCC values, first- and total-order indices, and p-values for each for measuring the sensitivity of the baseline value *x_b_* to model parameters. Parameters settings and methods of sensitivity analysis are identical to those in Table I.

| parameter | *w* | *a* | *k* | *q* | ω | *x*_0_ | *p* | σ | *n* |
| --- | --- | --- | --- | --- | --- | --- | --- | --- | --- |
| PRCC | -0.009 | -0.014 | -0.045 | -0.662* | -0.684* | 0.984* | -0.403* | 0.871* | 0.881* |
| *S_i_* | 0.000 | 0.000* | 0.000 | 0.021* | 0.022* | 0.758* | 0.006* | 0.089* | 0.092* |
| *S_Ti_* | 0.001 | 0.001 | 0.001 | 0.024* | 0.027* | 0.761* | 0.008* | 0.095* | 0.100* |

**References**

[A1] A. [Saltelli](http://eu.wiley.com/WileyCDA/Section/id-302479.html?query=A.+Saltelli), M. [Ratto](http://eu.wiley.com/WileyCDA/Section/id-302479.html?query=Marco+Ratto), T. [Andres](http://eu.wiley.com/WileyCDA/Section/id-302479.html?query=Terry+Andres), F. Campolongo,  [J. Cariboni, D. Gatelli](http://eu.wiley.com/WileyCDA/Section/id-302479.html?query=Debora+Gatelli), M. [Saisana](http://eu.wiley.com/WileyCDA/Section/id-302479.html?query=Michaela+Saisana), S. [Tarantola](http://eu.wiley.com/WileyCDA/Section/id-302479.html?query=Stefano+Tarantola), Global Sensitivity Analysis: The Primer, Wiley, New York, 2008.

[A2] S. Marino, I.B. Hogue, C.J. Ray, E. Kirschner, A methodology for performing global uncertainty and sensitivity analysis in systems biology, Journal of Theoretical Biology 254 (2008) 178-196.

[A3] A. Saltelli, J. Marivoet, Nonparametric statistics in sensitivity analysis for model output-a comparison of selected techniques, Reliability Engineering & System Safety 28 (1990) 229-253.

[A4] M. Ratto, A. Pagano, P. Young, State dependent parameter metamodeling and sensitivity analysis, Computer Physics Communications 177 (2007) 863-876.

[A5] S. Tarantola, D. Gatelli, T.A. Mara, [Random balance designs for the estimation of first order global sensitivity indices, Reliability Engineering & System Safety 91 (2006) 717-727.](http://apps.webofknowledge.com/full_record.do?product=WOS&search_mode=GeneralSearch&qid=1&SID=Q12SATjrGoXpVFUku5R&page=1&doc=4)
